# Medical Clinic, Containing Diseases of the Encephalon

**Published:** 1838-10-24

**Authors:** 


### BIBLIOGRAPHICAL NOTICE.

Medical Clinic, *Containing Diseases of the Encephalon.* By G. Andral. *Condensed and Translated by* D. Spillan, M. D. Philadelphia, 1838. 8vo. pp. 299.

In the last two numbers of the Select Medical Library, we are favoured with a republication of Spillan's translation of Andral's Clinique. This is one of the many valuable medical works, which would never have been republished in this country, except in the cheap form of a library edition. The demand for works of this description is limited to a small number of readers; the greater part of the profession are not sufficiently acquainted with their merits to take the trouble requisite to import them; nor is every physician in circumstances to pay the heavy expenses of purchasing an English edition. A few in every large city will of course read such works in the original language, but their number is too limited to exercise much influence upon the profession generally.

A part only of the work is yet published, that on the diseases of the brain and nervous system, that is, the organic diseases of the brain; as the work does not include the functional disorders, numerous and important as they are. But the kind of information which it gives, is not the less valuable on that account, for we are greatly in want, in this country, of those facilities for comparing the symptoms of diseases with the anatomical changes to which they correspond, that can only exist in large institutions specially devoted to particular diseases. On this account, the profession in all parts of the world, is obliged in a great degree, to depend upon the information received from the French capital; and pathological discoveries, for the most part, originate in France. Valuable, however, as the works upon pathology may be, they must be read with certain allowances, and in a proper spirit.

On the one hand, American physicians are apt to commit the error of supposing that the treatment in France, and particularly in French hospitals is extremely inert. Now, although there is some foundation for this notion, it is in many respects erroneous. All physicians who are conversant with large hospitals, in which there is no exclusion of particular diseases, on account of their incurability, know that a very large proportion of patients who die, enter the hospital after having exhausted their pecuniary resources, and having vainly tried all means of treatment within their reach. These patients, who may be in some measure relieved, cannot of course be cured; as they constitute a large proportion of those who are admitted into the French hospitals; very little active medicinal treatment is directed for them. Now, when this state of things is known to exist, such a course is not only consistent with sound reason, but becomes a sort of duty towards the patients. Their lot is only embittered by the more powerful remedies, which may seriously annoy them, without at all increasing their chances of recovery. One great, some say the greatest, advantage yet derived from pathology, is the power which it gives us of recognising these cases, and of avoiding useless and painful measures. The work of Dr. Andral is certainly one of the best for rendering the reader familiar with incurable diseases: the lesions of organs occurring in diseases are discussed in connexion with each case, which is given in sufficient detail for us to be sure of its nature.

There is another error often committed by readers of this class of works. It is that of supposing them perfect in their kind; they are disposed to quote the statements of an author as a proof that certain relations of disease are always to be viewed in the light in which they may have appeared to the earlier investigators. We must remember, that all faithful descriptions of disease include but a portion of the phenomena properly belonging to them, and that they must in time be superseded by the works of other observers who have commenced their investigations where their predecessors have terminated. The value of a really true work is not lost, but it is modified and is diminished by every successive addition to our knowledge.

The greater part of the observations of Dr. Andral were made some years since, others have been added more recently. The inquiries of physicians have been so successfully directed to the investigation of cerebral diseases, that a portion of his work is already incomplete, and requires additions to bring it up to the present state of the science. The author was aware that some additional observations on the usual diseases of children were requisite for comparison with those of adults, and they would certainly have added new value to the work. It contains, however, so much useful and practical matter, that we need scarcely regret that any thing should be wanting. Its deficiencies can be readily supplied from other sources, while the vast amount of practical observations which are carefully analyzed and compared with the facts collected from other quarters, could scarcely have been elsewhere obtained.

The work was prepared by the author, during the delivery of his course of lectures on the diseases of the brain, and in fact it contains most of the materials from which that course was prepared. The form is necessarily different from that of a course of lectures; less attractive, perhaps, but more convenient for practical reference. We well remember that it was a subject of deep regret with those who followed the course of Dr. Andral, a few years since, that no work then published was capable of supplying the materials which are necessary for a course of lectures, but which cannot well be introduced into the body of the course. The Medical Clinic not only supplies the deficiency, but retains much of the concise, nervous style, which is so characteristic of Andral's course on pathology.

We have one word more to say on the subject of the work, which is not susceptible of an analysis, but must be read and studied to be understood. It is the boldness, and we may say the propriety, of publishing cases which terminate fatally, rather than those which recover. Both fatal and successful cases have their use, but there is no question whatever that fatal cases give a more complete picture of a dangerous disorder than those which terminate favourably, and that they are even more useful in the therapeutic deductions which we glean from them. We may learn, at least, that a particular treatment did not cure the patient under the circumstances in which he was placed. If the disease proved fatal from causes within the control of the physician, he is bound to communicate them to the profession, that they may in future be avoided by others; if these circumstances were beyond his control, it is right to enumerate the various influences which may render a disease mortal.

Dr. Spillan has rendered so great a service to the profession, both in the British Islands and in America, by his laborious task, that we are not ungrateful enough to complain of the manner in which it is executed. The translation might have been written in better English, some errors might have been avoided, but they are not sufficiently grave or numerous materially to impair the general character of the work. The editor has in part corrected these errors, as the reader may perceive from the extract we make from Dr. Bell's preface to the present edition:—

"In this first American edition of the clinical experience of Andral on the Diseases of the Brain and its Meninges, we have made no change in the arrangement and narrative presented to us by the English translator, Dr. Spillan. We have, however, substituted, in many places, more idiomatic terms and phraseology for the gallicisms and French terms in which the English edition abounds. Some of them have probably escaped our notice; but they will not, it is believed, detract from the intrinsic and indisputable value of the work, which may safely be pronounced to be the very first order of merit. The accusation of diffuseness brought, not without reason, against some French works, cannot apply to the productions of Andral's pen. In the present instance, there is no toying with the reader's attention,—no attempt to amuse him by analogies or speculations; but there is, on the other hand, a continuous description and detail of a class of morbid alterations of structure and of symptoms growing out of them, a knowledge of which, by American physicians generally, is not, we fear, as accurate as could be desired in the actual state of modern pathology. The suggestions thrown out by Andral are couched in a spirit of philosophic caution, which are in singular contrast with the hasty and premature induction so observable in medical writings on both sides of the Atlantic. We have retained all the notes, and the additions on the spinal marrow and its membranes, introduced by the industrious and able translator, Dr. Spillan.

